# Poractant alfa (Curosurf^®^) increases phagocytosis of apoptotic neutrophils by alveolar macrophages in vivo

**DOI:** 10.1186/1465-9921-13-17

**Published:** 2012-03-09

**Authors:** Coen HMP Willems, Florian Urlichs, Silvia Seidenspinner, Steffen Kunzmann, Christian P Speer, Boris W Kramer

**Affiliations:** 1 Department of Pediatrics, School for Mental Health and Neuroscience (NUTRIM), School for Oncology and Developmental Biology (GROW), Maastricht University Medical Centre, Maastricht, The Netherlands; 2 University Children's Hospital, Würzburg, Germany; 3 Department of Pediatrics, Maastricht University Medical Center, Postbus 5800, 6202 AZ, Maastricht, The Netherlands

**Keywords:** Inflammation, Resolution, Anti inflammation, Drug therapy, Surfactant

## Abstract

**Background:**

Clearance of apoptotic neutrophils in the lung is an essential process to limit inflammation, since they could become a pro-inflammatory stimulus themselves. The clearance is partially mediated by alveolar macrophages, which phagocytose these apoptotic cells. The phagocytosis of apoptotic immune cells by monocytes in vitro has been shown to be augmented by several constituents of pulmonary surfactant, e.g. phospholipids and hydrophobic surfactant proteins. In this study, we assessed the influence of exogenous poractant alfa (Curosurf^®^) instillation on the in vivo phagocytosis of apoptotic neutrophils by alveolar macrophages.

**Methods:**

Poractant alfa (200 mg/kg) was instilled intratracheally in the lungs of three months old adult male C57/Black 6 mice, followed by apoptotic neutrophil instillation. Bronchoalveloar lavage was performed and alveolar macrophages and neutrophils were counted. Phagocytosis of apoptotic neutrophils was quantified by determining the number of apoptotic neutrophils per alveolar macrophages.

**Results:**

Exogenous surfactant increased the number of alveolar macrophages engulfing apoptotic neutrophils 2.6 fold. The phagocytosis of apoptotic neutrophils was increased in the presence of exogenous surfactant by a 4.7 fold increase in phagocytosed apoptotic neutrophils per alveolar macrophage.

**Conclusions:**

We conclude that the anti-inflammatory properties of surfactant therapy may be mediated in part by increased numbers of alveolar macrophages and increased phagocytosis of apoptotic neutrophils by alveolar macrophages.

## Background

Apoptosis and apoptotic cell clearance are recognized as important mechanisms in resolving inflammation, maintaining homeostasis and tissue remodeling, e.g. during ontogeny and repair [1]. Inefficient apoptotic cell clearance results in necrosis or cytolysis, which leads to the release of noxious cellular contents into surrounding tissues and consequently tissue damage and prolonged inflammation [1].

Efficient clearance of these apoptotic cells by phagocytes critically depends on a sequence of events. Firstly, the apoptotic cells undergo changes which target them for clearance, e.g. the loss of phospholipid asymmetry exposes phosphatidylserine on their cell surface [2]. Secondly, these changes of the cell surface need to be recognized by the phagocytes followed by their engulfment. This can be achieved through phagocyte receptors that interact directly with apoptotic cells and receptors that interact through intermediate soluble bridging molecules, like C1q and mannose-binding lectin, which attach to the surface of the apoptotic cells [3,4].

The efficient clearance of apoptotic cells and the resolution of inflammation are particularly important in organs like the lung, which are continuously exposed to the external environment. The detrimental effects of an inadequate response to inflammatory challenges in the lung can be observed in preterm infants. These preterm infants suffer from lung immaturity which is intimately linked to inflammatory events, in prenatal and immediate postnatal life [5]. These immature lungs are characterized by a lower surface area for gas exchange and a deficiency of pulmonary surfactant, which prevents alveolar collapse at end-expiration and is important for host defense. Together, these events trigger and contribute to the development of respiratory distress syndrome (RDS). RDS is still a leading cause of neonatal morbidity and mortality in the Western world. The incidence of RDS is consistently rising and inversely related to gestational age. In clinical practice, intratracheal administration of poractant alfa (Curosurf^®^) has shown efficacy in reducing the respiratory workload and improving the survival and outcome for premature infants suffering from severe RDS [6]. Poractant alfa consists of phospholipids, mainly dipalmitoylphosphatidylcholine, the primary surface-active agent of natural lung surfactant, and surfactant protein (SP)-B and SP-C, which facilitate spreading and adsorption of the surface-active agent at the air-alveolar interface [6]. However, the effects of poractant alfa are not limited to the biophysical effects of surface tension reduction. In vitro it has been shown to influence the phagocytic properties of human monocytes depending on the ingested cell type, e.g. micro-organisms or apoptotic cells [7,8]. Poractant alfa does however not contain the collectins SP-A or SP-D, which are well known for their functions in host defense and have been shown to increase the phagocytosis of apoptotic neutrophils by macrophages in vitro [9]. It was shown that severe RDS is linked to the activation of neutrophils [10] and can be caused or sustained by prolonged inflammation [5]. The observation that poractant alfa instillation significantly reduces morbidity and mortality in preterm infants suffering from severe RDS, together with the knowledge that RDS can be caused by prolonged inflammation, e.g. due to inefficient apoptotic cell clearance, raises the question if the resolution of inflammation can be exclusively addressed to SP-A and SP-D. We therefore hypothesized that the other constituents of pulmonary surfactant, present in poractant alfa, influence the resolution of the inflammatory response by regulating the phagocytosis of apoptotic cells in the lung.

To test the hypothesis that increased phagocytosis of apoptotic cells by alveolar macrophages may be part of the anti-inflammatory effects of poractant alfa therapy in vivo, we first studied its effect on phagocytosis of apoptotic neutrophils by alveolar macrophages in vitro and then in the lungs of adult mice. Poractant alfa or saline was instilled in the lungs of three month old C57/black mice followed by apoptotic neutrophils, i.e. annexin V positive and propidium iodide negative cells, or saline. The clearance of apoptotic neutrophils and recruitment of alveolar macrophages was assessed in bronchoalveolar lavage fluid (BALF). Furthermore, a phagocytosis index was determined from the ratio of alveolar macrophages participating in phagocytosis, i.e. myeloperoxidase (MPO) positive alveolar macrophages, and the average number of phagocytosed apoptotic neutrophils per alveolar macrophage, i.e. average number of MPO-positive vesicles per alveolar macrophage.

## Methods

### Reagents

Antibodies and the apoptosis detection kit were purchased from PharMingen (Mountain View, CA). All other reagents were purchased from Sigma Aldrich (St. Louis, MO).

### Myeloperoxidase staining

Myeloperoxidase (MPO) activity was assayed by measuring the H_2_O_2_-dependent oxidation of the chromogenic substrate 3-Amino-9-ethylcarbazole (AEC, Sigma Aldrich), which results in a red insoluble stain. This was followed by a hematoxylin background stain, resulting in an intense blue coloration of the nuclei.

### *In vitro *phagocytosis

Alveolar macrophages were isolated from mice by broncho-alveolar lavage [11]. Cells were washed twice with PBS at 4°C, centrifuged for 5 min at 400 × *g *at 4°C, and resuspended in culture media (DMEM) supplemented with 10% heat-inactivated fetal calf serum (Sigma Aldrich). After incubation at 37°C for 1 h, nonadherent cells were removed and plates were washed twice with media. The effect of poractant alfa on phagocytosis was tested in separate assays with apoptotic neutrophils and fluorescein-labeled beads (size 2 μm, Molecular Probes, Eugene, OR) (see below). All surfactant preparations were tested negative for endotoxin contamination with the *Limulus *amebocyte lysate assay (Sigma Aldrich). Phagocytosis of apoptotic neutrophils was tested by adding 2 × 10^5 ^apoptotic neutrophils to the adherent alveolar macrophages. Poractant alfa at a final concentration of 100 μg/mL phospholipids was added 15 min before the apoptotic neutrophils were added or the same volume of media in control experiments [8]. The alveolar macrophages and apoptotic neutrophils were incubated for 15 min at 37°C, washed with cold PBS containing 0.5 mM EDTA, and stained with MPO. Phagocytosis was evaluated by counting 100 macrophages per well. Similar experiments were performed with fluorescein-labeled beads (Molecular Probes). Phagocytosis was evaluated on an inverted microscope.

### Neutrophil isolation & flow cytometry

Neutrophils were isolated by Percoll gradient centrifugation from the whole blood of donors. Red blood cells were removed by osmotic lysis. Purity was assessed by staining MPO, which is expressed in neutrophils but not in monocytes. Apoptosis was induced in neutrophils by UV radiation (302 nm) for 30 min. Apoptosis was confirmed in > 85% of all cells by flow cytometry using fluorescently labeled annexin V to detect the phosphatidylserine expression on the cell surface of apoptotic cells and a propidium iodide counterstain (PharMingen). Preparations containing less than 8% necrotic cells were accepted. As a positive control, cell aliquots were incubated with camptothecin, a topisomerase I inhibitor that induces apoptosis, for 3 h at 37°C. Cells were kept on ice and analyzed on a FACSCalibur flow cytometer (Becton Dickinson, Mountain View, CA).

### Mice

The protocol was reviewed and approved by the local authorities. Three month old adult male C57/Black 6 mice (generous gift of Prof. Ulf Rapp, MSZ University Würzburg, Germany) were anesthetized with methoxyflurane and orally intubated with a 25-gauge animal-feeding needle. Poractant alfa (Curosurf^®^, generous gift of Chiesi Farmaceutici, Parma, Italy) in a dose of 200 mg/kg or the equivalent volume of saline (0.9% w/v NaCl) was instilled. Subsequently, 10^6 ^apoptotic neutrophils suspended in 100 μl of saline (only saline for controls) were injected via tracheal intubation in each mouse (n = 11 per group and timepoint).

The fate of the instilled apoptotic neutrophils was assessed after 15 min or 30 min respectively. A bronchoalveloar lavage was performed after the indicated time interval. Mice were deeply anesthetized with intraperitoneal pentobarbital sodium administration and exsanguinated by cutting the distal aorta (11). The thorax was opened, and a 20-gauge blunt needle was tied into the proximal trachea for alveolar lavage. Lungs were washed with 1 ml of 0.9% saline, which was infused and withdrawn by syringe three times. This lavage procedure was performed five times for each animal and pooled for analysis. Lavage fluid volumes were recorded and the lavages were immediately centrifuged for 15 min at 500 × *g *to spin down the cells. The pellets were resuspended in a defined volume of PBS and the cells were counted after staining with trypan blue (Scientific Products, McGaw Park, IL). Cytospins were stained for MPO activity to identify phagocytosed apoptotic neutrophils. Phagocytosis of apoptotic neutrophils was quantified by counting the number of MPO-positive vesicles in alveolar macrophages and determining the percentage of MPO-positive alveolar macrophages. A phagocytic index was calculated by multiplying the percentage of MPO-positive macrophages by the average number of phagocytosed vesicles.

### Data analysis

Results are given as mean ± SEM. Physiologic variables were analyzed using a two tailed t-test (for 2 groups) or one-way ANOVA with a Bonferroni post test (more than 2 groups). A *p*-value < 0.05 was considered significant. All statistical analyses were performed using the statistical software GraphPad Prism 5.0.

## Results

### *In vitro *phagocytosis

Alveolar macrophages increased phagocytosis of apoptotic neutrophils in vitro. The phagocytosis index increased from 20 ± 6 in control alveolar macrophages to 132 ± 39 in the poractant alfa incubated alveolar macrophages (*p *< 0.05). No effect of poractant alfa was detected in alveolar macrophages that were incubated with fluorescein-labeled beads. The phagocytosis index was 43 ± 7 without poractant alfa and 48 ± 9 with poractant alfa (not significant).

### Apoptotic neutrophils in BALF

**Figure 1:**
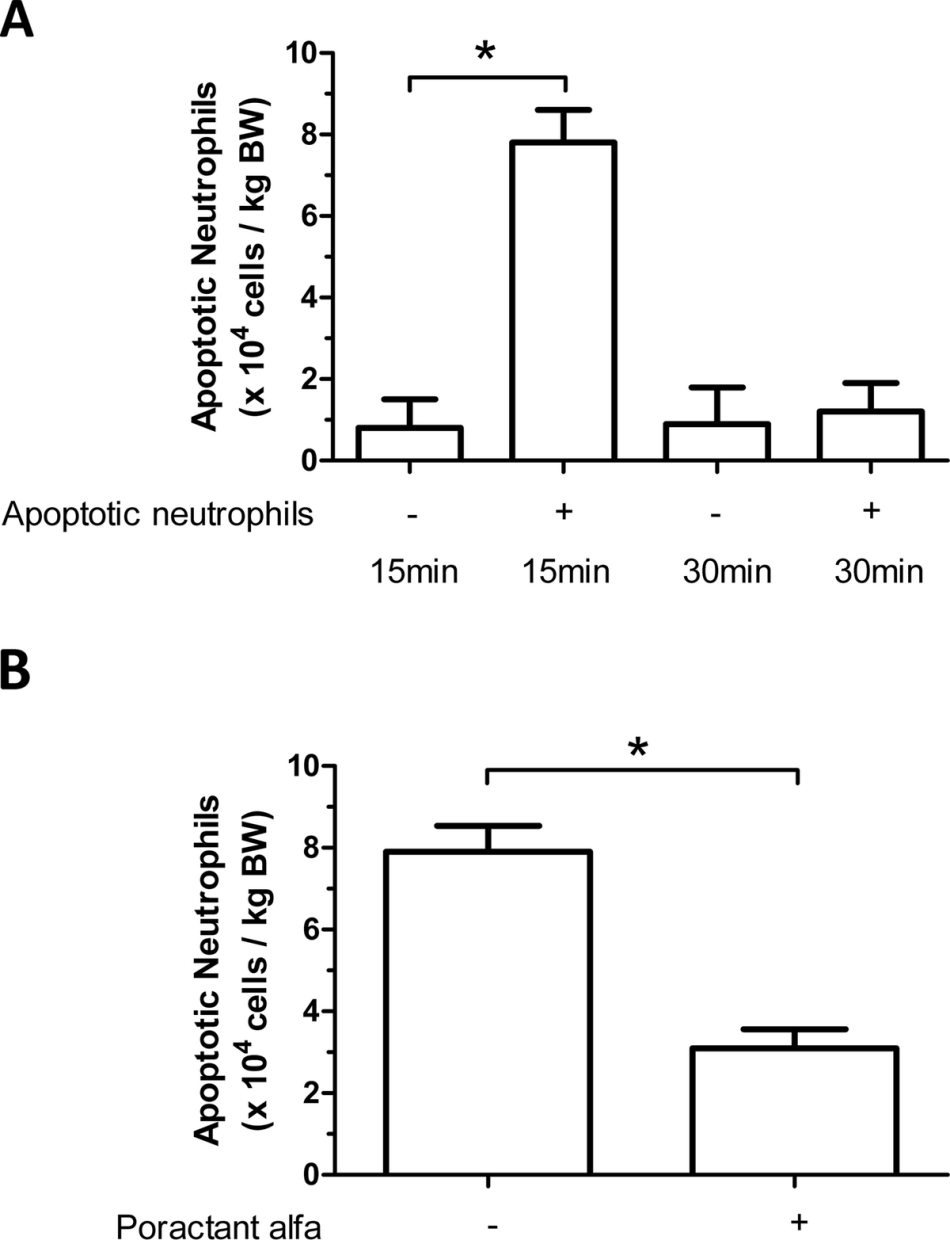
**A) Time course experiment showing the number of apoptotic neutrophils per kilogram body weight (BW) in BALF 15 or 30 minutes after intratracheal instillation of apoptotic neutrophils (+) or an equivalent volume of saline (-)**. B) The number of apoptotic neutrophils per kilogram body weight in BALF 15 minutes after administering 10^6 ^apoptotic neutrophils and subsequently instilling poractant alfa or an equivalent volume of saline. Results (n = 11 animals per group) are shown as mean ± SEM. Significant differences (*p *< 0.05) are marked by *.

A time course experiment (Figure 1a) revealed that the number of apoptotic neutrophils in BALF 15 minutes after instillation was elevated from 0.8 ± 0.7 × 10^4^/kg body weight (BW) in saline controls to 7.8 ± 0.8 × 10^4^/kg BW (*p *< 0.05) in treated mice. This marked increase in the number of apoptotic neutrophils dropped to background levels as soon as 30 minutes after instillation. The effect of exogenous surfactant instillation was therefore assessed 15 minutes after the instillation of apoptotic neutrophils. Poractant alfa reduced the number of apoptotic neutrophils detected in BALF from 7.9 ± 1.1 × 10^4^/kg BW in saline controls to 3.1 ± 0.8 × 10^4^/kg BW (*p *< 0.05) in treated mice (Figure 1b)

### Alveolar macrophages in BALF

**Figure 2:**
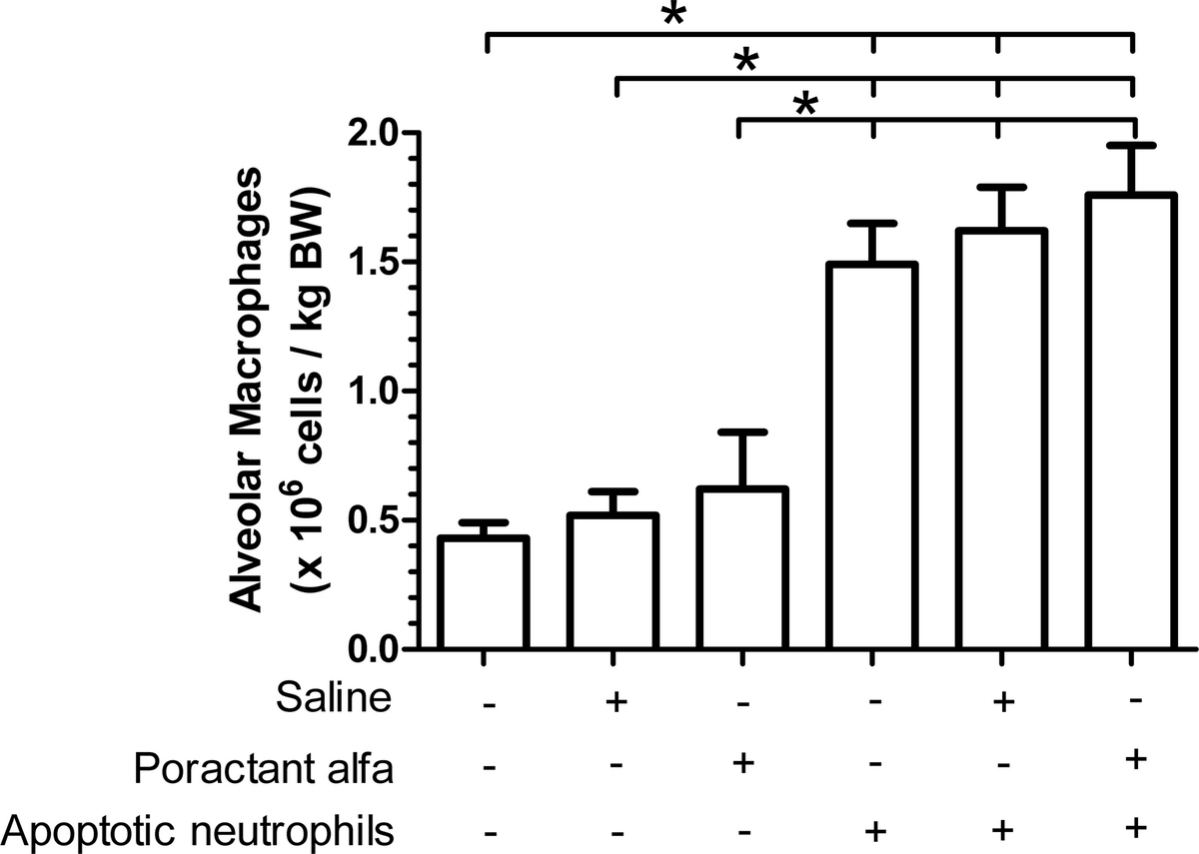
**The number of alveolar macrophages per kilogram of body weight in BALF of mice instilled with 10^6 ^apoptotic neutrophils or the equivalent volume of saline, receiving no treatment, saline or poractant alfa**. Results (n = 11 animals per group) are shown as mean ± SEM. Significant differences (*p *< 0.05) are marked by *.

The number of alveolar macrophages recovered from BALF in mice undergoing no intervention was 0.43 ± 0.06 × 10^6^/kg BW, which was unchanged by saline (0.52 ± 0.09 × 10^6^/kg BW) or poractant alfa (0.62 ± 0.22 × 10^6^/kg BW) instillation (Figure 2). After instillation of apoptotic neutrophils the number of alveolar macrophages increased to 1.49 ± 0.16 × 10^6^/kg BW (*p *< 0.05). Nor the treatment with saline (1.62 ± 0.17 × 10^6^/kg BW) or poractant alfa (1.76 ± 0.19 × 10^6^/kg BW) had any influence on the number of alveolar macrophages after apoptotic neutrophil instillation.

### Phagocytosis by alveolar macrophages

**Figure 3:**
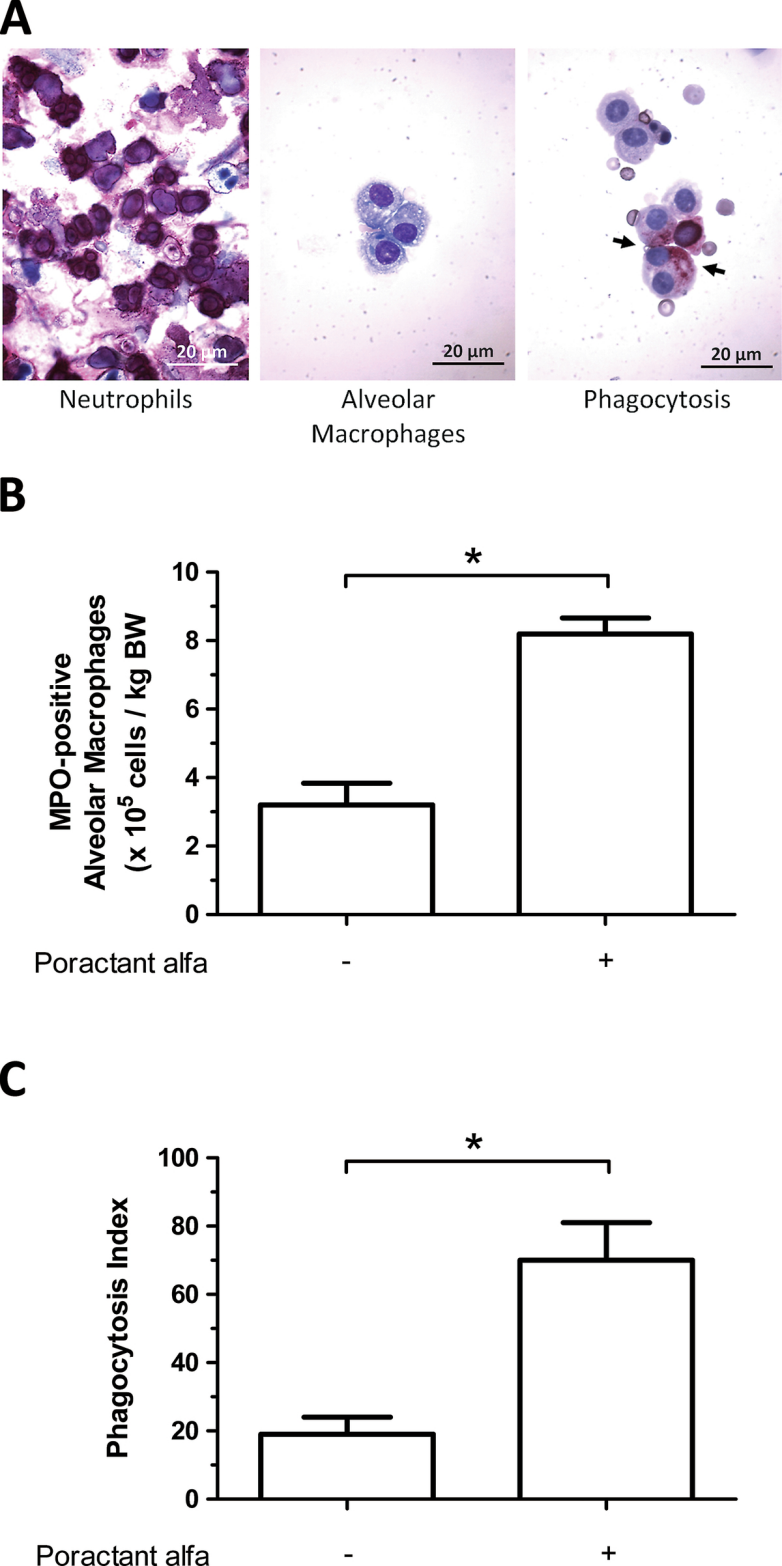
**A) Myeloperoxidase (MPO) staining of neutrophils before induction of apoptosis (left panel), alveolar macrophages in BALF from mice that received saline instillation (middle panel) and apoptotic neutrophils phagocytosed by alveolar macrophages in BALF from mice that received apoptotic neutrophils instillation (right panel)**. B) Number of MPO-positive alveolar macrophages per kilogram of body weight in BALF of mice instilled with 10^6 ^apoptotic neutrophils, receiving saline (-) or poractant alfa (+) treatment. C) Phagocytosis index of apoptotic neutrophils ingestion by alveolar macrophage in BALF of mice instilled with 10^6 ^apoptotic neutrophils, receiving saline (-) or poractant alfa treatment (+). The phagocytosis index was calculated by multiplying the percentage of MPO-positive alveolar macrophages with the average number of apoptotic neutrophils ingested per MPO-positive alveolar macrophage. Results (n = 11 animals per group) are shown as mean ± SEM. Significant differences (*p *< 0.05) are marked by *.

The number of macrophages ingesting apoptotic neutrophils and the amount of apoptotic neutrophils phagocytosed by each macrophage was assessed by MPO staining (Figure 3a). After instillation of apoptotic neutrophils, the treatment with poractant alfa increased the number of MPO-positive macrophages to 8.2 ± 0.8 × 10^5^/kg BW (*p *< 0.05) compared to saline treated controls (3.2 ± 1.1 × 10^5^/kg BW, Figure 3b). The phagocytosis index (Figure 3c) after instillation of apoptotic neutrophils increased from 19 ± 5 after saline injection to 70 ± 11 after poractant alfa instillation (*p *< 0.05 vs saline injection).

## Discussion

In this paper we demonstrated for the first time that poractant alfa, an exogenous porcine derived lung surfactant, increased the clearance of apoptotic neutrophils in vivo. This was the result of both an increased number of alveolar macrophages engulfing apoptotic neutrophils and a higher number of phagocytosed apoptotic neutrophils per alveolar macrophage.

Both apoptosis and the removal of apoptotic cells are vital during development, maintaining homeostasis and resolving inflammation in the lung. These processes are all involved in the development and resolution of RDS. Nowadays exogenous surfactant (e.g. poractant alfa) replacement therapy and ventilation are standard of care for infants suffering from severe RDS in the Western world. The reduction of surface tension and workload through surfactant therapy greatly reduces the morbidity and mortality [6]. However, the close association of RDS with inflammation suggests the beneficial modes of action of surfactant go beyond the reduction of workload. Indeed, there is a vast array of research on the immunomodulatory functions of surfactant [12]. Several studies have reported immunomodulatory functions of poractant alfa or its lipid constituents on various levels of the inflammatory response. For instance, it was shown to impair the phagocytosis of microorganisms by monocytes [7], impair the respiratory burst of monocytes and neutrophils exposed to inflammatory stimuli [13,14] and modulate the release of pro- and anti-inflammatory cytokines [7,15-19]. The phagocytosis of apoptotic cells under influence of poractant alfa has also been studied in vitro [8]. This study revealed that poractant alfa enhanced the phagocytosis of apoptotic cells by monocytes.

We have now tested this observation in adult mice in vivo to confirm our in vitro findings. Specifically, this study provides evidence that apoptotic neutrophils are cleared efficiently by recruiting alveolar macrophages as soon as 15 minutes after instillation. This fast response indicates these macrophages resided in close apposition to the alveolar unit, e.g. the interstitial space. In the control animals the number of apoptotic neutrophils returned to background levels within 30 minutes. Poractant alfa enhanced the clearance of apoptotic neutrophils, significantly lowering their levels within 15 minutes, without altering the amount of recruited macrophages. MPO staining of cells in BALF revealed that the enhanced clearance of apoptotic neutrophils was the result of an increased ratio of macrophages participating in the clearance (MPO-positive macrophages) and an elevated activity of these macrophages (phagocytosis index). This ultimately resulted in a vast reduction of the number of apoptotic neutrophils in the lungs resolving the inflammation [20-22].

The experiments showed that only a low percentage of instilled apoptotic neutrophils (~3%) could be recovered in bronchoalveolar lavage fluid and in the mobilized alveolar macrophages. This is partially explained by the low recovery of alveolar macrophages by bronchoalveolar lavage techniques that can only mobilize approximately 30% of the alveolar macrophages [23]. Besides the low rate of recovery of alveolar macrophages as an inherent technical limitation, the kinetics of alveolar macrophages in the trafficking to and from the blood vessels and interstitium cannot be estimated in this model. Mechanisms involved in the observed effects remain elusive since most studies on the immunomodulatory functions of surfactant, including the phagocytosis of apoptotic cells, investigated the role of the hydrophilic surfactant proteins SP-A and SP-D, which are not present in poractant alfa. A comprehensive study by Gardai et al. [24] shed light on the apparent conflicting data from some of these diverse studies on these immunomodulatory properties of SP-A and SP-D, by pointing out that the various domains of these proteins have opposing functions. They revealed the collagenous tail domain was responsible for phagocytosis of pathogens and enhancing the production of pro-inflammatory mediators by alveolar macrophages, while the head domain, which recognizes and opsonizes pathogens, inhibited the production of these mediators. The last decade the involvement of collectins SP-A and SP-D in the clearance of apoptotic neutrophils has also gained attention, but has to date not been fully elucidated [25]. These studies have however shown that for the clearance of apoptotic cells the various domains of these proteins seem to fulfill different roles. Schagat et al. revealed that the lectin domain was not involved in the phagocytosis of apoptotic neutrophils, but the carbohydrate moieties of SP-A were [25]. However, it appeared that these were involved in binding alveolar macrophages rather than apoptotic neutrophils. An involvement of the lipid binding capabilities of SP-A in recognizing apoptotic cells could not be ruled out. Similar to Gardai et al. [24], another study showed that during apoptotic cell clearance SP-A and SP-D bind macrophages through their collagenous domain by engaging with the CD91/calreticulin receptor complex [26]. However, these effects could not be completely inhibited by blocking CD91/calreticulin, which could indicate redundant receptors and mechanisms involved in apoptotic cell clearance. Overall, this redundancy in receptors, mechanisms and bridging molecules hampers the unambiguous investigation of events in vivo. Additionally, recent evidence suggests that these collectins differently engage binding with viable, early and late apoptotic cells [27-29]. This is further complicated by the fact that divergent results have been observed for resident and recruited alveolar macrophages [30]. A more recent study by Janssen et al. [31] shed some light on some of these divergent results. They showed that the removal of alveolar macrophages improved the phagocytic activity to levels observed for macrophages of other origin and that the exposure to SP-A and SP-D reduced their phagocytic activity. They hypothesized that a tonic interaction of SP-A and SP-D with SIRPα suppressed alveolar macrophage phagocytic function. This could also explain our observations, that an instillation of poractant alfa improves phagocytosis, i.e. the lipids in surfactant compete with SIRPα in binding the head domain of these collectins, thereby lifting their suppressive effects on phagocyte function. However, the suggested mechanisms require further investigation, for instance by evaluating the influence of supplementing the surfactant preparations with SP-A and/or SP-D in the future.

One limitation of our study was that adult animals were used as a model, while surfactant administration is currently limited to the neonatal period. However, this was necessary since we demonstrated earlier that the total number of alveolar macrophages is too small in the premature lung for current detection methods [32]. Alveolar macrophages differentiate in the fetal lung late in gestation from blood derived monocytes [33]. The induction of alveolar macrophages can be induced in utero by proinflammatory stimuli causing chorioamnionitis [32], however these experiments could not be performed in preterm mice.

In summary, exogenous porcine derived lung surfactant poractant alfa increased the phagocytosis and clearance of apoptotic neutrophils by alveolar macrophages in vivo. Surfactant therapy may therefore possess anti-inflammatory properties in vivo, by enhancing the resolution of the inflammation and ultimately protecting the pulmonary tissue from damaging inflammatory responses. The extrapolation of these effects to immature lungs needs to be done with caution. We have however previously shown in vitro that similar effects could be observed with blood derived monocytes, which may be the prevailing cell type in the immature lung [32].

## Conclusion

This study shows that exogenous surfactant therapy may possess anti-inflammatory properties in vivo. The anti-inflammatory properties of surfactant therapy were in part mediated by increased numbers of alveolar macrophages and increased phagocytosis of apoptotic neutrophils by alveolar macrophages.

## Abbreviations

SP: Surfactant Protein; MPO: Myeloperoxidase; BALF: Bronchoalveolar Lavage Fluid; BW: Body Weight.

## Competing interests

The authors declare that they have no competing interests.

## Authors' contributions

BK initiated and designed the study. CW, FU, SS, SK, CS and BK were responsible for the conduction of the study and data acquisition, FU and BK conducted the animal experiments, CW and BK were responsible for data analysis and the interpretation of data. All authors have read and approved the final manuscript.
